# Community and healthcare worker contributions to malaria prevention: a culturally tailored behaviour change approach in Jozini, South Africa

**DOI:** 10.1186/s12936-026-05943-8

**Published:** 2026-05-27

**Authors:** Ramokone Ednah Baloyi, Sanele Ngcobo, Taneshka Kruger, Christiaan de Jager

**Affiliations:** 1 School of Health Systems and Public Health, Faculty of Health Sciences, University of Pretoria, Dr Savage Rd, Prinshof 349-Jr, Pretoria, 0084 South Africa; 2 National Department of Health, 1112 Voortrekker Rd, Pretoria Townlands 351-Jr, Pretoria, 0187 South Africa; 3 Department of Family Medicine, University of Pretoria, 7th Floor 7-28 H, 31 Bophelo Road, Pretoria, 0084 South Africa; 4 UP Institute for Sustainable Malaria Control (UP ISMC), University of Pretoria Private Bag X323, Arcadia, 0007 South Africa

**Keywords:** Malaria elimination, Malaria prevention, Knowledge, Attitude and practices, Social Behavioural Change Communication, South Africa

## Abstract

**Background:**

According to the national strategic plan, South Africa aims to eliminate malaria by 2030. Only three out of 9 provinces are malaria endemic. These are Mpumalanga, Limpopo and the north–eastern parts of KwaZulu-Natal provinces. It is important to investigate community and healthcare worker perceptions regarding malaria prevention, control and elimination interventions to enable development of a Social Behavioural Change Communication (SBCC) strategy for Jozini local municipality to complement the National Strategic Plan 2025–2030.

**Methods:**

A cross-sectional mixed-method study was conducted in six communities and their respective healthcare facilities in Jozini local municipality, KwaZulu-Natal, during 2025. A structured questionnaire was administered to 384 community participants using a systematic household sampling. In addition, semi-structured interviews were conducted with 15 healthcare workers selected purposively. Quantitative data were analysed using descriptive statistics, Chi-square (χ^2^) tests (p < 0.05) and Fisher’s exact test (p < 0.05) where expected cell counts were less than 5, while qualitative responses were analysed thematically.

**Results:**

Three hundred and eighty-four community members and 15 healthcare workers were interviewed. Community respondents (median age 36 years; 72% female) showed high malaria awareness (96%), with 92% correctly identifying mosquito bites as the main transmission route. Overall, 97% had high knowledge, 79% positive attitudes, and 64% good practices. Knowledge was significantly associated with age (χ^2^ = 9.61, p = 0.01) but not with gender, education, employment status, or residence duration, and knowledge level was not significantly associated with attitudes or practices (p > 0.05). Healthcare workers showed strong technical knowledge of malaria transmission and prevention, and most reported involvement in diagnosis, treatment, and community outreach activities. However, qualitative findings highlight challenges related to irregular training, staffing gaps, and shortages of diagnostic tools and commodities.

**Conclusions:**

The study found that although awareness of malaria is generally high in Jozini local municipality, misconceptions and inconsistent prevention practices remain. Community health workers are key information sources, but barriers such as poverty, cultural beliefs, and limited resources affect prevention efforts. Healthcare providers are knowledgeable but face operational challenges. These findings underscore the need for targeted SBCC interventions to support malaria control under South Africa’s NSP 2025–2030.

## Background

Malaria remains a public health challenge affecting marginalised poorer communities, with global estimates reported to be 282 million new cases and 610 000 related deaths in 2024 [1]. The World Health Organisation has reported that most of the world’s malaria cases are reported from Sub-Saharan Africa, with Democratic Republic of Congo, Uganda, and Mozambique reported to have the highest incidence [1]. Mozambique, one of the top malaria reporting countries borders South Africa along its north-eastern provinces, where malaria transmission remains high [2]. This border region is a key source of imported malaria cases into South Africa, posing a challenge to the country’s elimination efforts [2]. This puts the country at risk for cross border malaria, as it is a key source of imported malaria cases [2]. This risk in importation might slow down the country’s quest to eliminate malaria by 2030 [2, 3].

In South Africa, malaria transmission remains concentrated in three endemic provinces (KwaZulu-Natal, Mpumalanga and Limpopo), with uMkhanyakude district in northern KwaZulu-Natal reporting persistent local transmission. [3, 4] Malaria surveillance data indicate that Jozini Local Municipality has reported approximately 3,841 malaria cases over the past 5 years, representing an estimated annual prevalence of 0.8–1%. This highlights the continued public health need of targeted elimination strategies in this area. [4]

Despite the risk of importation, South Africa has made significant progress in reducing its malaria burden [3]. This reduction in malaria burden is marked by South Africa being among the four front line countries in the Southern African Development Community (SADC) Elimination 8 region identified [3, 5]. These are countries with the potential to eliminate malaria by 2030 [3]. KwaZulu-Natal in particular, out of three districts that are endemic in the country, has reached subnational elimination in King Cetshwayo district and has not reported any indigenous malaria cases in Zulu Land district for the past four malaria seasons [6, 7]. While uMkhanyakude continues to experience indigenous malaria, progress has been made in reducing case numbers, reinforcing the importance of continued surveillance, prevention, and community-focused interventions in Jozini local municipality. [6, 7]

Although KwaZulu-Natal is the front runner to achieve malaria elimination status in South Africa, in depth effort, particularly in Jozini local municipality in understanding behavioural and socio-economic factors that influences malaria prevention and healthcare seeking behaviours need to be prioritised. It has been cited that community beliefs, perceptions and traditional practices play a role in how malaria interventions in communities are responded to [8]. This in turn impacts the effectiveness of intervention measures employed by malaria workers and efforts to strengthen timeous health-seeking behaviours to avert deaths and ongoing transmission.

Successes in addressing health-related behaviours, promoting positive practices and strengthening community engagements has been linked to the implementation of Social and Behavioural Change Communication (SBCC) strategies [8–11]. Despite this, the effectiveness of SBCC strategies is reliant on how culturally relevant they are and how they are tailored to respond to local needs [8–11]. Misconceptions about malaria transmission, traditional healing practices, socio-economic barriers to access to health care are all factors that can influence both individual and community level malaria response effectiveness [12, 13]. Understanding these factors is key in developing targeted SBCC strategies that not only complement the South African National malaria Strategic plan 2025–2030 (NSP) but, also resonates with the target population and ultimately enhance intervention uptake.

Healthcare workers are critical in malaria prevention and treatment [14, 15]. Their knowledge, attitudes and communication strategies can have an impact on community behaviours, perceptions and engagement with malaria services. Understanding their knowledge, attitudes and practices(KAP) will provide insights into healthcare delivery successes, challenges, community interactions and gaps in malaria healthcare provision among residents of Jozini local municipality [15]. A previous study in Zambia emphasised the importance of healthcare workers’ perspectives and practices in the identification of operational challenges and trust issues that influence the effectiveness of case management responses by the malaria programme. [16]

Understanding both community and healthcare worker KAP will provide key insights in the development of a targeted evidence based SBCC approach geared towards accelerating malaria elimination in Jozini local municipality.

Previous studies demonstrated how interventions designed without a proper understanding of local perceptions, behaviours, and socio-cultural dynamics fall short in achieving meaningful community engagement or sustained behavioural change [17, 18]. This emphasises the importance of KAP studies in identifying specific gaps to address [17]. A study conducted in Eswatini demonstrated that insights into local perceptions of malaria transmission and treatment are essential for informing elimination strategies. [17]

This study aimed to explore and understand the KAP related to malaria prevention and treatment-seeking behaviours among Jozini local municipality residents, with additional focus on the roles of healthcare workers. The contextualisation will inform the development of a culturally appropriate and community-responsive SBCC strategy that aligns with the NSP 2025–2030 objectives.

## Methods

### Study design

A cross-sectional mixed-methods design, which combined quantitative a household-based questionnaire administered to community participants and qualitative interviews among healthcare workers involved in malaria prevention and control. The study was conducted between March and May of 2025, in Jozini local municipality.

### Study setting

**Fig. 1:**
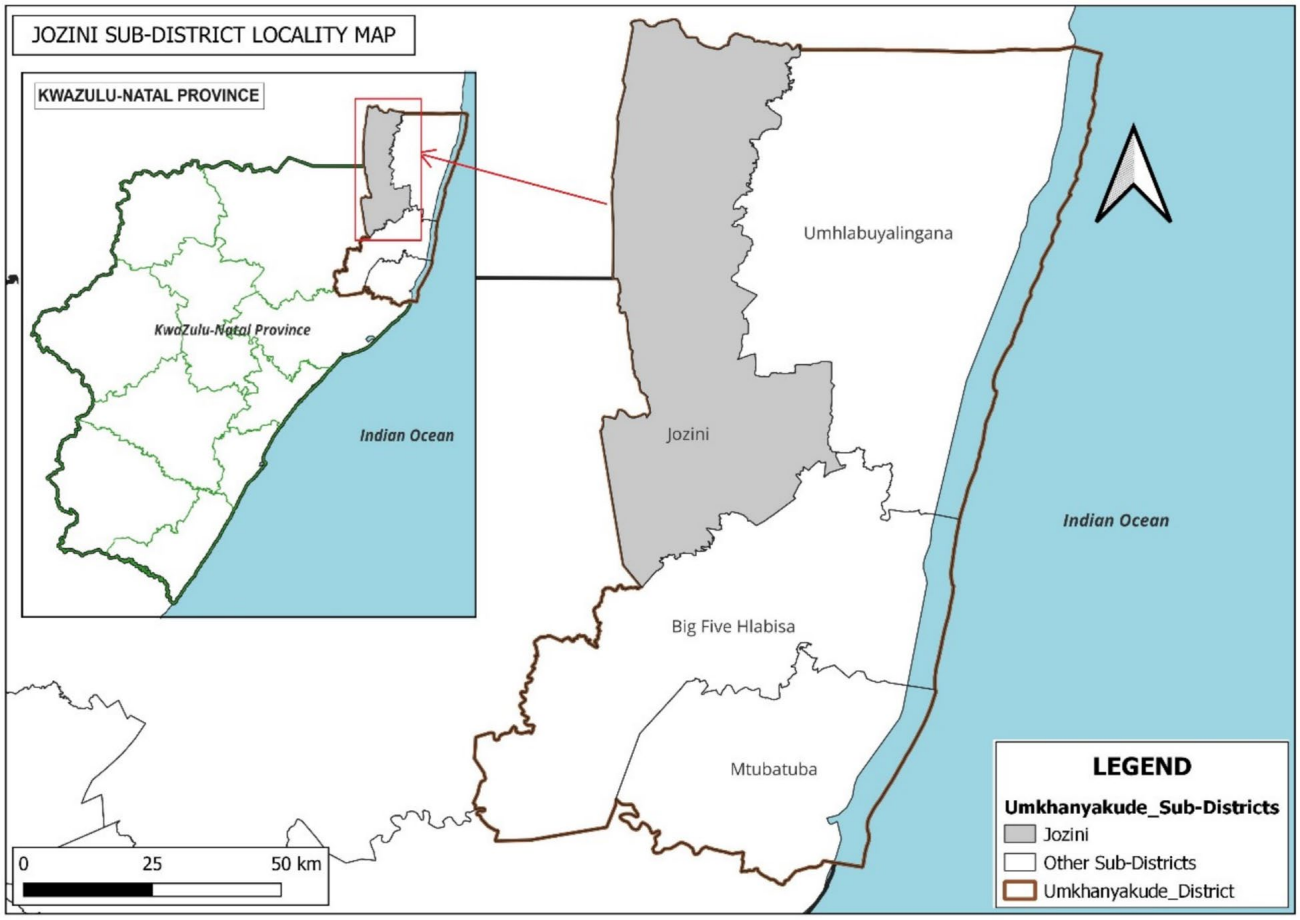
Map of KwaZulu-Natal District Municipalities, South Africa, showing the Jozini study area. [7]

This study was conducted in Jozini local municipality, situated in uMkhanyakude District in the northern part of KwaZulu-Natal Province, South Africa (Fig. 1. The local municipality is characterised by rural and semi-rural settlements, with subtropical climate marked by warm temperatures and seasonal rainfall conditions favourable for malaria transmission [19, 20]. The area includes environmental features such as the Jozini Dam and surrounding low-lying areas, which may provide suitable habitats for mosquito breeding. [19, 20]

The local municipality includes six localities that continue to report local malaria transmission. According to the most recent South African census data, the total population of the municipality is approximately 199 153 people [21]. The demographic profile is relatively youthful, with individuals aged 15–56 years comprising the majority of the population [21]. The sex ratio is 85.8, indicating fewer men than women. [21]

The local economy is largely based on subsistence agriculture, livestock farming, small-scale trading, and informal economic activities, with many households relying on agriculture and social support grants as primary sources of livelihood. [20]

### Study population

The study population comprised community members aged 18 years and older who were residing in the selected localities as well as healthcare workers involved in malaria prevention and control in these areas.

Healthcare workers included nurses, Environmental Health Practitioners (EHPs) and Malaria Surveillance Agents (MSAs).

### Inclusion criteria

The study included female and male adults aged 18 years and older, who were permanent residents for at least one year and were able to provide informed consent.

Eligible healthcare workers were those involved in malaria prevention and control, with a minimum of one year of experience in their current role.

### Exclusion criteria

Community members younger than 18 years, and those who had not resided in the municipality for at least one year, were excluded.

Among healthcare workers, pharmacists, pharmacist assistants, administrative staff and data capturers were excluded, as they were not directly responsible for malaria prevention and control activities.

### Sampling strategy

#### Community based study

A multistage sampling design was used, involving purposive selection of sections based on malaria transmission levels. One low-transmission section (0.1– < 1 local-indigenous case per 1000 population at risk) and one moderate-transmission section (≥ 1 local-indigenous case per 1000 population at risk) were selected in each locality [22]. Two sections from each of the six localities (12 Sections in total) were selected. This was followed by systematic random sampling of households within selected sections.

The community leaders (izinduna) and Ward Councillors in the six selected localities were engaged through scheduled meetings. During these meetings, the purpose, objectives, and procedures of the study were explained, and their support was sought to mobilise residents from the selected sections. Households were then systematically selected using the malaria program's pre-assigned numbering system, employing a random sampling method. Trained field assistants, consisting of MSAs, visited these households, provided detailed explanations about the study, confirmed eligibility, obtained informed consent from participants and administered the questionnaire. If there was no person who met the inclusion criteria, the house was excluded from the study.

#### Healthcare worker study

Purposive sampling was used to recruit ten MSAs and EHPs from the malaria programme in Jozini local municipality. Recruitment involved engaging the Malaria Programme Manager to identify eligible participants and securing their consent to participate. The same was done for facility-based healthcare workers (nurses), where facility managers were engaged to identify eligible participants to take part in the study.

Five facilities were included in the study, with one facility selected from each section and one healthcare worker recruited from each facility.

This multi-step approach ensured an ethical, inclusive, and representative recruitment. Further to this, it ensured a holistic exploration of local cultural and social factors that influence malaria prevention and healthcare-seeking behaviour among the population of Jozini local municipality.

### Sample size

#### Community based study

For the community, the sample size was calculated from the total population of Jozini local municipality, which was 199,153. The proportions of KAP regarding malaria transmission in this setting were unknown; a 50% proportion was assumed to ensure the maximum size. A 50% prevalence assumption was used because the true prevalence of malaria-related knowledge, attitudes, and practices in the study population was unknown. Using 50% ensures the maximum sample size and adequate statistical precision in cross-sectional surveys.

The sample size was calculated using Stata Version 19, assuming a population size of 199 153, 50% prevalence, 95% confidence interval and 5% precision. A 90% response rate was assumed, which came to a final sample size of 384 people. The calculated sample size provided adequate precision to estimate KAP proportions at 95% confidence level.

#### Healthcare worker study

Fifteen healthcare workers were recruited using purposive sampling. This component was primarily qualitative and was designed to capture insights into healthcare workers’ operational experiences, perceptions and challenges related to malaria prevention and control. Although the sample size was small, it reflects the limited number of personnel directly involved in malaria programme implementation in the selected facilities and programme structures and was considered adequate for generating contextual insights.

## Measurements

### Data collection

#### Community based study

For the community-based study, English and IsiZulu-speaking field assistants were trained in data collection techniques through questionnaires. The training sessions occurred in English and the EHPs translated it into isiZulu. Further training was conducted by the EHPs to the field assistants who administered the questionnaire. The questionnaire was adapted from a tool previously used during a community KAP study on malaria in Eswatini in 2009 [18]. The questionnaire was divided into five sections: (i) respondent demographics, (ii) knowledge, (iii) attitudes, (iv) social practices and (v) treatment-seeking behaviour related to malaria transmission and disease. The questionnaire included open-ended, partially closed, and closed questions.

#### Healthcare worker study

For the health system aspect, interviews were conducted using a semi-structured questionnaire. The interviews were conducted in English, focusing on the roles, behaviours and perceptions of the healthcare workers.

### Variables

#### Community based study

The demographic characteristics of the community participants included locality, age, gender, type of occupation, education level and the number of years the participants have been residing in the municipality. KAP scores were given according to the participant’s responses to the questionnaire. The explanatory variables were the demographic characteristics of the participants, and the outcome variables were the KAP scores.

#### Healthcare worker study

The healthcare worker’s demographic characteristics included the health facility/ locality they work in, age, gender, type of occupation and number of years working on malaria prevention and control.

### Data management

Data for both the community and healthcare workers were collected using paper-based questionnaires. These were captured into google forms and exported in Excel (Microsoft Corp.,USA) for cleaning. Data quality assessment included for completeness, missing data, logical inconsistencies and duplicates. Cleaned datasets were analysed using Stata version 19 (StataCorp, USA) for quantitative data and ATLAS.ti version 25.0.1 for qualitative data.

### Data analysis: community participants

#### Community based study

##### Descriptive analysis

To describe the community participants’ characteristics, numerical data was summarised using medians and Interquartile Ranges (IQR). Categorical variables were summarised using frequency and percentages. Descriptive statistics and 95% confidence intervals were used to summarise community participants’ KAP scores.

##### KAP scoring

Knowledge questions (n = 3) assessed community participants’ understanding of malaria transmission, prevention, and recognition of symptoms. Correct responses were scored as 1, and incorrect responses as 0; total knowledge scores ranged from 0–3. Participants scoring ≥ 2 were classified as having high knowledge.

Attitude questions (n = 3) used a combination of Likert scales and categorical responses to assess perceived seriousness of malaria, timeliness of treatment-seeking, and perceived effectiveness of preventive measures. Positive responses were scored as 1, negative responses as 0; total attitude scores ranged from 0–3. Participants scoring ≥ 2 were categorized as having a positive attitude.

Practice questions (n = 7) assessed participants’ use of preventive measures, frequency of bed net use, prompt treatment-seeking, and avoidance of traditional remedies. Good practices were scored as 1 and poor practices as 0, with total scores ranging from 0–7. Participants scoring ≥ 4 were classified as having good malaria prevention practices.

For each KAP domain, median IQR were calculated, and participants were categorized into high/low knowledge, positive/negative attitude, and good/poor practice for descriptive analysis.

##### Inferential analysis

Associations between KAP outcomes and participants’ characteristics were analysed using the Chi-square (χ^2^) test, while Fisher’s exact test was used where expected cell counts were less than five. A p-value < 0.05 was considered statistically significant.

#### Healthcare worker study

##### Descriptive analysis

Healthcare worker characteristics were summarised using medians (IQR) for continuous variables and frequencies with percentages for categorical variables. KAP scores for healthcare workers were analysed descriptively using medians, IQR, frequencies, and 95% confidence intervals.

##### Qualitative data analysis

Open-ended responses from healthcare workers were analysed using thematic analysis within ATLAS.ti 25.0.1. This was guided by deductive-inducive approach. The deductive component was informed by the KAP framework, while inductive coding allowed for the emergence of unanticipated themes grounded in the data.

Codes were generated inductively and grouped into broader themes related to malaria knowledge, clinical practice in malaria case management, perceptions of malaria intervention tools, and health system gaps affecting malaria control.

All open-ended responses were imported into ATLAS.ti 25.0.1 for data management and analysis. Each respondent’s set of responses constituted a single primary document. Survey questions were grouped within ATLAS.ti 25.0.1 according to conceptual domains (knowledge, attitudes, practices, and system-level factors) to facilitate structured retrieval and comparison.

An initial codebook was developed prior to coding, based on the KAP framework and study objectives. This included parent codes for KAP, and capacity/system factors. During the first cycle of coding, responses were coded line-by-line to meaning units. New codes were added inductively where participant responses did not adequately fit pre-existing categories.

In the second coding cycle, codes were reviewed, refined, and merged where conceptual overlap existed. Code definitions were clarified, and consistent application was ensured across all documents. ATLAS.ti 25.0.1 code manager and code-document tables were used to monitor code frequency and distribution.

### Ethical considerations

Ethical approval was obtained from the University of Pretoria’s Faculty of Health Sciences Research Ethics Committee (Ref: 762/2024), the uMkhanyakude District Ethics Committee, and the National Health Research Database (Ref: KZ_202502_019). All participants provided written informed consent. Participation was voluntary and anonymous, with data coded using unique identifiers. Completed questionnaires were securely stored, and electronic data were kept on a password-protected computer to ensure confidentiality.

## Results

### Sociodemographic characteristics of study participants

A total of 384 community members and 15 healthcare workers participated in the study. Community participants had a median age of 36 years (IQR: 29–48) and were predominantly female (72%). More than half had completed secondary education, and unemployment was high (84%) (Table 1). Healthcare Workers had a mean age of 36.4 years and were mostly female (60%). The majority held higher education qualifications (80%) and had more than five years of malaria-related work experience (73%), Table 1.

**Table 1:** Demographic characteristics of study participants in the selected study households in the Jozini Local municipality, South Africa, 2025

Community based study (N = 384)	
Age	N (%)
*Median (IQR)	36 (29–48)
18–35	160 (41.7)
36–50	126 (32.8)
51–65	69 (18.0)
> 65	29 (7.6)
Gender	
Female	278 (72.4)
Male	106 (27.6)
Education	
No formal	60 (15.6)
Primary	97 (25.3)
Secondary	203 (52.9)
Higher	24 (6.3)
**Occupation	
Unemployed	323 (84.1)
Agriculture	11 (2.9)
Education	4 (1.0)
Health/Traditional	2 (0.5)
Service	12 (3.1)
Labor/Transport	5 (1.3)
Security	3 (0.8)
Self-employed	5 (1.3)
Pensioner	14 (3.6)
Student	3 (0.8)
Number of Years living in Jozini local Municipality	
1–5 years	19 (4.9)
> 5 years	365 (95.1)
Healthcare worker-based study (N = 15)	
Age	
Mean (range)	36.4 (23–59)
Gender	
Female	9 (60.0)
Male	6 (40.0)
Education	
Secondary/Diploma	3 (20.0)
Higher	12 (80.0)
Year in your Current role	
1–5 years	4 (26.7)
> 5 years	11 (73.3)
Occupation	
Nurse	5 (33.3)
Malaria Surveillance Agent	5 (33.3)
Environmental Health Practitioner	4 (26.7)
Entomologist	1 (6.7)

^*^IQR interquartile range

^**^Service includes Builder, Cook, Creche Teacher, Driver, Field Ranger, Funeral

### Parlor worker, helper, plumber, seamstress, truck driver

#### Community based study responses

Table 2 shows the responses from the community-based study. Knowledge of malaria was high among community participants. Most reported knowing what malaria is (96.0%) and correctly identified mosquito bites as the mode of transmission (92.0%). Community malaria interventions were reported by 59.1%. Community health workers were the primary information source (96.9%), followed by radio (27.1%), while social networks contributed minimally (4.7%). Nearly half of participants perceived malaria as not serious (47.9%), although most indicated they would seek treatment immediately if symptomatic (95.8%).

**Table 2:** Summary of participant characteristics and malaria KAP indicators with 95% confidence intervals

	Indicator	% (N)	95% CI
Knowledge	Community Healthcare Workers (CHWs) as information source	96.9%(372)	95.1–98.6
	Know what malaria is	96.0% (367)	92.3–96.8
	Identify mosquito bites as transmission	92.0% (353)	88.7–94.5
	Community malaria interventions present	59.1% (227)	54.0–64.1
	Radio as information source	27.1% (104)	22.6–31.5
	Social networks as information source	4.7% (18)	2.6–6.8
Attitudes	Would seek treatment immediately	95.8% (368)	93.3–97.4
	Malaria not serious	47.9% (184)	42.9–52.9
	Very serious	18.0% (69)	14.4–22.1
	Moderately serious	15.9% (61)	12.2–19.5
	Slightly serious	14.6% (56)	11.0–18.1
	Extremely serious	3.4% (13)	1.6–5.2
Practices	Sleep under bed nets	84.4% (324)	80.4–87.9
	Use one method only	60.2% (231)	55.1–65.2
	Use insect repellents	50.5% (194)	45.4–55.6
	Other preventive measures nightly	50.3% (193)	45.1–55.4
	Never use other measures	27.3% (105)	22.9–32.1
	Take malaria prophylaxis	26.0% (100)	21.7–30.7
	Use all three methods	21.1% (81)	17.1–25.7
	Use two methods	18.8% (72)	15.0–23.3
Treatment	Seek treatment immediately	76.6% (294)	72.0–80.7
	Never consult healers	70.3% (270)	65.5–74.8
	Rarely consult healers	21.4% (82)	17.4–25.8
	Seek treatment 1–2 days	20.1% (77)	16.2–24.4
	Sometimes consult healers	5.5% (21)	3.4–8.2
	Seek treatment 3 + days	2.9% (11)	1.4–5.1
	Often consult healers	2.9% (11)	1.4–5.1

Preventive practices were common: 84.4% slept under bed nets, 50.5% used insect repellents, and 26.0% used prophylaxis. Sixty percent used only one method, 18.8% used two, and 21.1% used all three measures. Half reported using additional preventive measures nightly (50.3%), while 27.3% never did so. Most participants sought treatment promptly, with 76.6% seeking care immediately and 20.1% within one to two days. Traditional healer consultation was uncommon, with 70.3% reporting that healers were never consulted for malaria and only 2.9% reporting frequent consultation. Median scores reflected generally good KAP levels: knowledge (median 3, IQR 2–3), attitudes (median 2, IQR 2–3), and practices (median 4, IQR 3–5). Overall, 96.9% demonstrated high malaria knowledge, 78.9% positive attitudes, and 63.8% good prevention practices.

##### Influence of knowledge on participants’ attitude and practices on malaria prevention and control

Attitude did not differ significantly by knowledge level (χ^2^ = 1.21, *p* = 0.47); most participants with either low or high knowledge reported positive attitudes Table 3. Similarly, malaria-related practices showed no significant association with knowledge (χ^2^ = 1.02, *p* = 0.31). Although slightly more participants with low knowledge reported poor practices compared with those with high knowledge, the overall distribution indicates that knowledge level did not significantly influence practice outcomes, Table 3.

**Table 3:** Association of knowledge on participants’ attitude and practices towards malaria prevention and control (Fisher’s exact test)

Characteristic	Knowledge		Fisher’s exact statistic	p-value
	Low N = 12	High N = 372		
Attitude			1.21	0.47
Negative	1(1.2)	80(98.8)		
Positive	11(3.6)	292(96.4)		
Practices			1.02	0.31
Poor	6(4.3)	133(95.7)		
Good	6(2.5)	239(97.5)		

##### Association between socio-demographic characteristics and participants’ level of KAP on malaria prevention

A significant association was found between age and malaria knowledge (χ^2^ = 9.61, p = 0.01), with low knowledge more common among participants aged 18–35 years (6.3%) compared to older age groups (≤ 2.1%). Overall, malaria knowledge was high, with 96.9% of participants classified as knowledgeable. Knowledge did not differ significantly by gender, education level, employment status, or length of residence (all p > 0.05), Table 4.

**Table 4:** Test of association between socio-demographic characteristics and KAP practices towards malaria prevention and control

Characteristic	Knowledge		Fisher’s exact statistic	p-value	Attitude		Fisher’s exact statistic	p-value	Practices		Fisher’s exact statistic	p-value
	Low	High			Negative	Positive			Poor	Good		
	N = 12	N = 372			N = 81	N = 303			N = 139	N = 245		
Age (years)			9.61	0.01			1.39	0.50			1.72	0.42
18–35	10(6.3)	150(93.7)			34(21.3)	126(78.7)			64(40.0)	96(60.0)		
36–50	0(0.0)	126(100.0)			30(23.8)	96(76.2)			42(33.3)	84(66.7)		
51 +	2(2.1)	96(97.9)			17(17.4)	81(82.6)			33(33.7)	65(66.3)		
Gender			0.04	1.00			2.49	0.12			3.29	0.07
Female	9(3.2)	269(96.8)			53(19.1)	225(80.9)			93(33.5)	185(66.5)		
Male	3(2.8)	103(97.2)			28(26.4)	78(73.6)			46(43.4)	60(56.6)		
Education			0.49	0.84			2.43	0.29			2.42	0.29
No formal	2(3.3)	58(96.7)			16(26.7)	44(73.3)			24(40.0)	36(60.0)		
Primary	2(2.1)	95(97.9)			23(23.7)	74(76.3)			40(41.2)	57(58.8)		
Secondary/Higher	8(3.5)	219(96.5)			42(18.5)	185(81.5)			75(33.1)	152(66.9)		
Employment status			0.83	0.41			1.59	0.21			0.45	0.51
Unemployed	9(2.8)	315(97.2)			72(22.2)	252(77.8)			115(35.5)	209(64.5)		
Employed	3(5.0)	57(95.0)			9(15.0)	51(85.0)			24(40.0)	36(60.0)		
Number of Years living in Jozini local municipality			0.30	0.46			0.34	0.78			1.99	0.22
1–5 years	1(5.3)	18(94.7)			3(15.8)	16(84.2)			4(21.1)	15(78.9)		
> 5 years	11(3.1)	354(96.9)			78(21.4)	287(78.6)			135(36.9)	230(63.1)		

No socio-demographic variables were significantly associated with attitudes toward malaria prevention (all p > 0.05). Although negative attitudes were more frequent among men than women (26.4% vs. 19.1%), this difference was not statistically significant.

Similarly, malaria prevention practices showed no significant associations with socio-demographic characteristics (all p > 0.05). Poor practices were most common among younger participants and males, but these differences did not reach statistical significance, Table 4.

#### Healthcare worker study responses

##### Knowledge of malaria transmission and prevention

Healthcare workers demonstrated substantial biomedical knowledge regarding malaria transmission, predominantly identifying mosquito bites as the principal mode of infection. Participants described transmission in technically accurate terms, for example:

“Malaria is passed on to people by an infected mosquito when it bites them.” (P01–M–45–54).

“Because I know that it is caused by an Anopheles mosquito that carries the parasite.” (P02–M–35–44).

Participants also expressed confidence in established preventive measures, particularly Indoor Residual Spraying (IRS):

“They are effective; in fact they are the ones that reduce malaria cases in our area.” (P01–M–45–54).

Despite this strong provider knowledge, respondents highlighted ongoing community misconceptions and inconsistent use of prevention methods:

“They work well in theory, but adherence depends on whether people actually use the nets every night.” (P11–F–25–34).

#### Attitudes toward malaria prevention and treatment

Overall, participants expressed favourable perceptions of existing malaria interventions, describing them as effective when properly implemented. Many healthcare workers conveyed confidence in the technical value of preventive strategies such as IRS and insecticide-treated nets (ITNs). For example:

“They are effective; in fact they are the ones that reduce malaria cases in our area.” (P01–M–45–54).

“These methods have proven results; when we implement them properly, we see fewer cases.” (P09–F–45–54).

However, frustration emerged regarding inconsistent community uptake and correct use of preventive measures. Several participants emphasised that intervention success depended largely on community cooperation and adherence:

“The methods work, but people do not always use the nets properly.” (P11–F–25–34).

“They work well in theory, but it depends on whether people actually sleep under the nets every night.” (P11–F–25–34).

“Mostly they are effective, but education is needed because some people don’t follow the advice.” (P12–F–35–44).

Healthcare workers’ confidence in malaria control efforts was therefore tempered by challenges in influencing community behaviours, suggesting that attitudes are shaped not only by knowledge of intervention efficacy but also by contextual implementation realities.

#### Roles and practices in malaria service provision

Healthcare workers described multifaceted roles encompassing diagnosis, treatment, health education, and community outreach. Respondents emphasized engagement with households and communities as integral to malaria control, reflecting an expansion beyond clinical responsibilities.

“My role includes testing patients, giving treatment, and teaching families how to prevent malaria.” (P03–F–35–44).

“We don’t only treat; we also go into the community to educate people and check if they are using their nets.” (P08–F–45–54).

“Sometimes we visit households to follow up cases and talk to families about prevention.” (P05–M–35–44).

This theme underscores the breadth of responsibilities shouldered by frontline workers and highlights the importance of supporting non-clinical activities that contribute to prevention.

#### Training, resource constraints, and work challenges

While some participants reported receiving malaria-related training, many highlighted gaps in the frequency, comprehensiveness, or timeliness of such training. Several respondents expressed a need for refresher courses to remain updated on evolving guidelines and protocols:

“We have had training before, but not regularly. We need updates so that we stay current.” (P06–F–35–44)

“Sometimes the training is once-off, and after that there is no follow-up.” (P10–M–45–54)

Limited availability of essential resources, including diagnostic tools, antimalarial medicines, and preventive commodities such as bed nets, further constrained service delivery. Participants frequently described how these shortages undermined their ability to provide effective care:

“I feel prepared, but sometimes we do not have enough supplies to do the work properly.” (P04–F–25–34).

“There are days when test kits or medication run out, and it delays helping patients.” (P07–M–35–44).

These system-level challenges negatively affected both service quality and worker morale, illustrating how structural constraints shape frontline performance despite adequate knowledge and commitment. ATLAS.ti co-occurrence analysis revealed that training deficiencies and resource shortages were strongly associated with reported work challenges, indicating that individual capacity is significantly influenced by systemic factors.

#### Suggested improvements and community engagement strategies

Participants provided a range of recommendations aimed at strengthening malaria prevention, treatment, and community engagement in Jozini local municipality. These recommendations focused on training and capacity building for healthcare workers, improved community education and communication strategies, enhanced availability of resources and diagnostic tools, strengthened health system support, and better coordination across sectors. Table 5 summarises the key recommendation areas and presents supporting verbatim quotations that reflect participants’ perspectives and experiences.

**Table 5:** Key recommendation areas and supporting verbatim quotations that reflect participants’ perspectives and experiences

Analytic category	Participant recommendation	Supporting verbatim quotations
Training and capacity building	Increased frequency and continuity of training	“More training, the frequency of the training can be more.” (P01–M–40–49)
		“More regular training.” (P02–M–40–49)
	Updated malaria treatment and case management training	“Enhance training for healthcare workers on updated malaria treatment guidelines and case management.” (P06–F–30–39)
Community education and health promotion	Expanded malaria education and awareness	“Increase community education and awareness about malaria transmission, prevention, and the importance of early treatment.” (P03–M–40–49)
	Improved packaging of health promotion messages	“The daily health promotion message should be packaged differently – to be sticky and appealing to the community.” (P10–F–40–49)
Resources and service delivery	Consistent availability of commodities and transport	“Consistent supply of commodities e.g. IRS items. including in non-endemic district” (P05–M–50–59)
Human resources and system strengthening	Addressing staffing gaps and stock management	“Replacement of retired staff and fill all vacant post in the programme.” (P07–F–40–49)
Community engagement strategies	Engagement of community leaders and structures	“Organize community dialogue sessions where local leaders, elders, and health workers discuss malaria issues openly with residents.” (P03–M–40–49)
Communication and innovation	Use of digital platforms for malaria messaging	“Using digital platforms / social media where people spend more of their time.” (P05–M–50–59)
Tools, diagnostics, and infrastructure	Strengthening diagnostic and laboratory capacity	“Highly sensitive RDT that will detect all types of malaria parasite even at their earliest stage.” (P05–M–50–59)
Coordination and governance	Improved multisectoral coordination	“Stronger coordination with other health sectors and NGOs for comprehensive support.” (P11–M–40–49)

## Discussion

This study provides baseline information on malaria-related KAP at both the community level and among healthcare providers in South Africa. The findings aim to contribute to the development of a SBCC strategy to support the country’s NSP 2025–2030. The findings from this study reveal a mixed picture that shows promising awareness in some areas but also highlights gaps that need to be addressed to improve the overall effectiveness of malaria elimination strategies in the municipality.

Overall, community members demonstrated good knowledge of malaria, with nearly all participants having heard of the disease prior to the study [18, 23]. Consistent with findings from previous KAP studies in KwaZulu-Natal and Eswatini, most respondents correctly identified mosquito bites as the primary mode of transmission. However, a small number of misconceptions remain, including beliefs that malaria is spread through dirty water or bad air, indicating the continued need for targeted health education to address knowledge gaps. [17, 21]

CHWs were identified as the primary and most trusted source of malaria information, followed by radio, with minimal reliance on social networks. This pattern is consistent with evidence from Odisha, India, where high awareness coexisted with persistent misconceptions, highlighting the continued influence of frontline and community-based information sources [24]. Perceptions of malaria risk were low among community members despite high levels of awareness. Nearly half of participants did not regard malaria as a serious health problem, indicating a disconnect between knowledge and perceived threat. Similar patterns have been reported in Zanzibar and Ghana, where declining or seasonal transmission was associated with reduced risk perception and weaker preventive behaviours. [25, 26]

Most participants could name effective prevention methods, especially the use of bed nets and insect repellents. The consistent use of these were low. Almost half of the participants reported to sleep under a bed net every night and over a quarter said they never use one. Similar to this, only half used insect repellents regularly. The gaps identified between knowledge and practice appear to be influenced by practical barriers, such as hot weather during the night making it uncomfortable to sleep under bed nets, the cost of repellents, or limited indoor protection when people sleep outdoors [27]. Similar patterns arise globally: in North India and the Solomon Islands, technical access did not guarantee use, especially when nets were uncomfortable or difficult to hang [24, 26]. In regions like Tanzania, Zanzibar, and Ghana, barriers such as heat, inadequate net size, and discomfort were cited as reasons for low adherence. [28]

This disconnect between knowledge and practice suggests that behavioural and structural barriers, rather than awareness alone, may influence prevention behaviours, highlighting the importance of context-sensitive SBCC strategies.

The participants acknowledged the effectiveness of bed nets but noted their impracticality during hot weather or when sleeping outside. Others spoke about the costs of buying insecticides or repellents, especially in a community where unemployment is high. These insights suggest that malaria prevention strategies must be adapted to the realities of people’s daily lives and socioeconomic status. Efforts to increase bed net use, for example, should include consideration of local sleeping patterns, climate, and economic constraints.

### Healthcare-seeking behaviours and use of traditional remedies

Encouragingly, almost all participants reported that they seek treatment immediately when they or a family member shows symptoms of malaria. This aligns with studies in other African regions where over 97% of symptomatic individuals sought care at health facilities [29]. The public health system remains the main point of care, with most of the participants seeking help from clinics or hospitals. Nonetheless, some participants reported utilising traditional treatments, indicating that traditional beliefs and practices continue to have an impact on certain people.

There were acknowledgments from some participants that people in their communities consult traditional healers for malaria. A study conducted in Tanzania, found that traditional healers remain influential in managing febrile illness, even when biomedical care is available.^32^ This highlights the need to engage with traditional healers as part of broader SBCC strategies, rather than dismissing them outright.

### Healthcare workers’ knowledge and capacity

Healthcare workers interviewed in this study showed a strong foundational knowledge of malaria, including transmission modes and prevention methods. Most considered malaria to be a serious concern in their community and felt adequately prepared to carry out their roles. The effectiveness of these workers was often limited by systemic challenges.

The healthcare workforce who participated in the study were largely experienced, with most having been in their roles for over five years. Despite this, gaps in training were evident, with only two-thirds who had received formal training on malaria. The workers strongly emphasised the need for regular refresher training to keep up with evolving guidelines.

Additional to training needs, the workers cited logistical barriers such as lack of transportation, poor supervision, and difficulty accessing remote areas, especially during rainy seasons. These directly affected the delivery of timely and consistent malaria services to vulnerable communities.

The healthcare workers who participated in the study reported to be using a combination of door-to-door visits, clinic talks, and community meetings to engage residents. Many also worked in partnership with schools and traditional leaders. These multifaceted approaches are promising and reflect an understanding that community buy-in is essential for sustained behaviour change.

However, participants also reported on opportunities for improvement, such as introducing school-based education programs and using SMS reminders during peak transmission seasons. These suggestions are not only feasible but could also significantly enhance reach and timeliness of messaging.

## Limitations

The study used a cross-sectional design, which restricts the ability to draw causal inferences or assess changes in KAP over time. Secondly, there might have been over-reported positive behaviours such as bed net usage or timely healthcare seeking as the findings are based on self-reported data. Recall bias may also have affected the accuracy of responses, particularly for questions requiring retrospective reporting.

Although the sample size for community participants was robust, the healthcare provider component involved only 15 individuals, which limits the generalisability of those findings. Future studies would benefit from a mixed-methods approach, larger provider samples, and a longitudinal design to better understand trends and drivers of malaria prevention and treatment behaviour. Although in the community-based study, some associations were observed in multivariable analysis, the small number of participants in certain outcome categories may have limited the stability of estimates. The small number of participants classified as having low knowledge limited statistical power and should be considered when interpreting association analyses.

## Conclusions

The study reports findings of the KAP of the Jozini local municipality community, a malaria endemic area that is close to malaria elimination. Some key findings were that while the knowledge of malaria in Jozini local municipality is relatively high, especially among healthcare providers, there are still factors that continue to hinder the effectiveness of prevention and treatment strategies. Among community members, low risk perception and underuse of preventative tools limit the impact of available interventions. Healthcare workers demonstrated strong knowledge but face systemic challenges such as irregular training, stockouts, and difficulties ensuring community adherence. These factors include low risk perception, economic barriers, underuse of preventive tools, and logistical challenges in healthcare delivery.

There is a need to address these barriers through a dual approach: reinforcing community engagement to promote consistent preventive practices and strengthening health system support to enable effective service delivery by healthcare workers. By integrating the voices of both residents and community healthcare workers, this study offers a clear roadmap for strengthening the SBCC component of malaria elimination efforts in the region.

## Data Availability

Data is available upon request.

## Abbreviations

CHW: Community Health Worker
EHP: Environmental Health Practitioner
IEC: Information, Education, and Communication
IRS: Indoor Residual Spraying
ITN: Insecticide-Treated Net
KAP: Knowledge, Attitudes, and Practices
MSA: Malaria Surveillance Agent
NSP: National Strategic Plan (for Malaria Elimination)
RDT: Rapid Diagnostic Test
SADC: Southern African Development Community
SBCC: Social and Behavioural Change Communication
WHO: World Health Organization

## References

[1] World Health Organization. World malaria report 2025. Geneva: World Health Organization; 2025.

[2] Maharaj R, Morris N, Seocharan I, Kruger P, Moonasar D, Mabuza A, et al. The feasibility of malaria elimination in South Africa. Malar J. 2012;11:423. 10.1186/1475-2875-11-423. 23253091 10.1186/1475-2875-11-423 PMC3573969

[3] Southern African Development Community. Situation and response analysis report on malaria in the SADC region. Gaborone: Southern African Development Community; 2009. Available from: www.sadc.int. Accessed 10 Mar 2026.

[4] Mberikunashe J. Southern African Development Community malaria scorecard. Gaborone: Southern African Development Community; 2023.

[5] Mabona M, Baloyi RE, Shandukani MB, Mogadime M, Wanjala S, Raman J. A review of malaria trends in South Africa for the 2022–2023 malaria season based on the Notifiable Medical Conditions Surveillance System. Public Health Bull South Afr. 2024.

[6] National Department of Health South Africa. KwaZulu-Natal map stratified by locality. Pretoria: DHIS2-MIS; 2024.

[7] Bose DL, Hundal A, Singh S, Singh S, Seth K, Hadi S ul, et al. Evidence and gap map report: social and behavior change communication interventions for strengthening HIV prevention and research among adolescent girls and young women in low- and middle-income countries. Campbell Syst Rev. 2023;19(1). 10.1002/cl2.1297. 10.1002/cl2.1297 PMC9831290 36911864

[8] Storey D, Obregon R. The role of social and behavioral change communication to address inequities and disparities in public health: reflecting on themes from the third international social and behavior change summit. J Health Commun. 2023;28:1–4. 10.1080/10810730.2023.2257940. 38146159 10.1080/10810730.2023.2257940

[9] Koniz-Booher P, Rogers B, Moreaux M. SBCC in the Sahel: a landscape assessment of nutrition and hygiene social and behavior change communication in Niger and Burkina Faso. Washington: SPRING Project; 2014. Available from: www.spring-nutrition.org. Accessed 10 Mar 2026.

[10] Solihin O, Mogot Y, Madonna M. Social behavior communication change for handling infectious diseases in Bandung. Available from: https://www.researchgate.net/publication/362113671. Accessed 10 Mar 2026.

[11] Udosen EE, Henshaw AA, Ogri EU. Stakeholders advocacy and the campaign against malaria epidemic in Cross River State: a social change perspective. LWATI J Contemp Res. 2019.

[12] RBM Partnership to End Malaria. The strategic framework for malaria social and behaviour change communication 2018–2030. Geneva: RBM Partnership to End Malaria; 2018.

[13] Brückner K, Emberger-Klein A, Menrad K. How do emotions influence healthy food choice? Investigating an extended framework of the social-cognitive theory. Br Food J. 2024;126(13):486–503. 10.1108/BFJ-01-2024-0105.

[14] Casella A, Monroe A, Toso M, Hunter G, Underwood C, Pillai R, et al. Understanding psychosocial determinants of malaria behaviours in low-transmission settings: a scoping review. Malar J. 2024;23. 10.1186/s12936-023-04831-9. 10.1186/s12936-023-04831-9 PMC10782749 38200574

[15] Chanda P, Hamainza B, Moonga HB, Chalwe V, Pagnoni F. Community case management of malaria using ACT and RDT in two districts in Zambia: achieving high adherence to test results using community health workers. Malar J. 2011;10:158. 10.1186/1475-2875-10-158. 21651827 10.1186/1475-2875-10-158 PMC3121653

[16] Lopez AR, Brown CA. Knowledge, attitudes and practices regarding malaria prevention and control in communities in the Eastern Region, Ghana, 2020. PLoS One. 2023;18(8). 10.1371/journal.pone.0290822. 10.1371/journal.pone.0290822 PMC10468076 37647322

[17] Hlongwana KW, Mabaso MLH, Kunene S, Govender D, Maharaj R. Community knowledge, attitudes and practices on malaria in Swaziland: a country earmarked for malaria elimination. Malar J. 2009;8:29. 10.1186/1475-2875-8-29. 19228387 10.1186/1475-2875-8-29 PMC2649151

[18] Statistics South Africa. Mid-year population estimates 2025. Pretoria: Statistics South Africa; 2025. Available from: https://www.statssa.gov.za. Accessed 10 Mar 2026.

[19] National Department of Health South Africa. Malaria elimination strategic plan for South Africa 2019–2023. Pretoria: National Department of Health; 2019.

[20] Manana PN, Kuonza L, Musekiwa A, Mpangane HD, Koekemoer LL. Knowledge, attitudes and practices on malaria transmission in Mamfene, KwaZulu-Natal Province, South Africa 2015. BMC Public Health. 2018. 10.1186/s12889-017-4583-2. 10.1186/s12889-017-4583-2 PMC5520288 28728572

[21] Das A, Gupta RD, Friedman J, Pradhan MM, Mohapatra CC, Sandhibigraha D. Community perceptions on malaria and care-seeking practices in endemic Indian settings: policy implications for the malaria control programme. Malar J. 2013;12:39. 10.1186/1475-2875-12-39. 23360508 10.1186/1475-2875-12-39 PMC3570348

[22] Beer N, Ali AS, Eskilsson H, Jansson A, Abdul-Kadir FM, Rotllant-Estelrich G, et al. A qualitative study on caretakers’ perceived need of bed nets after reduced malaria transmission in Zanzibar, Tanzania. BMC Public Health. 2012;12:606. 10.1186/1471-2458-12-606. 22863188 10.1186/1471-2458-12-606 PMC3438043

[23] Ahorlu CS, Adongo P, Koenker H, Zigirumugabe S, Sika-Bright S, Koka E, et al. Understanding the gap between access and use: barriers and facilitators to insecticide-treated net use in Ghana. Malar J. 2019;18:409. 10.1186/s12936-019-3051-0. 31831004 10.1186/s12936-019-3051-0 PMC6909499

[24] Raman J, Morris N, Frean J, Brooke B, Blumberg L, Kruger P, et al. Reviewing South Africa’s malaria elimination strategy (2012–2018): progress, challenges and priorities. Malar J. 2016. 10.1186/s12936-016-1497-x. 27567642 10.1186/s12936-016-1497-x PMC5002155

[25] Zerdo Z, Anthierens S, Van Geertruyden JP, Massebo F, Biresaw G, Shewangizaw M, et al. Implementation of a malaria prevention education intervention in southern Ethiopia: a qualitative evaluation. BMC Public Health. 2022;22:1. 10.1186/s12889-022-14200-x. 36151537 10.1186/s12889-022-14200-x PMC9508754

[26] Khairy S, Al-Surimi K, Ali A, Shubily HM, Al Walaan N, Househ M, et al. Knowledge, attitude and practice about malaria in south-western Saudi Arabia: a household-based cross-sectional survey. J Infect Public Health. 2017;10(5):499–506. 10.1016/j.jiph.2016.09.021. 28254460 10.1016/j.jiph.2016.09.021

[27] Hutchinson P, Zulliger R, Butts JK, Candrinho B, Saifodine A, Eisele TP, et al. Interpersonal communication, cultural norms, and community perceptions associated with care-seeking for fever among children under age five in Magoé district, Mozambique. Malar J. 2023. 10.1186/s12936-023-04689-x. 37735394 10.1186/s12936-023-04689-x PMC10515048

[28] Bachan EG, Abu J, Adokiya MN, Tobin-West C. Effectiveness of malaria control interventions in resource-limited countries: a systematic review. J Interv Epidemiol Public Health. 2025. 10.37432/jieph-d-25-00060.

[29] Makundi EA, Malebo HM, Mhame P, Kitua AY, Warsame M. Role of traditional healers in the management of severe malaria among children below five years of age: the case of Kilosa and Handeni districts, Tanzania. Malar J. 2006;5:58. 10.1186/1475-2875-5-58. 16848889 10.1186/1475-2875-5-58 PMC1540433

